# Association between micronutrient deficiency and acute respiratory infections in healthy adults: a systematic review of observational studies

**DOI:** 10.1186/s12937-019-0507-6

**Published:** 2019-11-30

**Authors:** Min Xian Wang, Jiayun Koh, Junxiong Pang

**Affiliations:** Centre for Infectious Disease Epidemiology and Research, Saw Swee Hock School of Public Health, National University of Singapore, 12 Science Drive 2, Singapore, 117549 Republic of Singapore

**Keywords:** Micronutrients, Deficiency, Respiratory infections, Adults, Vitamin D

## Abstract

**Background:**

Acute respiratory infections (ARI), including the common cold causes significant morbidity and economical losses globally. Micronutrient deficiency may increase ARI incidence risk and its associated duration and severity among healthy adults, but evidence are inconclusive. This study aims to systematically review all observations on the association between single micronutrient deficiency and ARI incidence, duration and severity in healthy adults.

**Methods:**

Systematic literature searches were conducted in PubMed, Cochrane Library, Embase and Scopus databases. Eligible studies were assessed for the reporting and methodological quality. Adjusted summary statistics with their relevant 95% confidence intervals or interquartile ranges were extracted for the outcomes of interest.

**Results:**

The literature search identified 423 unique studies. Of which, only eight studies were eligible and included in the final review. Only vitamin D deficiency (VDD) was observed among these eight studies. There were no eligible studies that focused on the association between other single micronutrient deficiency and ARI. The review found mixed observations on ARI incidence, and a lack of evidence on its associated severity to conclude the association between VDD and these outcomes. However, existing evidence consistently suggested that VDD is likely to lead to longer ARI duration (median duration in days: deficient group, 4 to 13; non-deficient groups, 2 to 8).

**Conclusion:**

This review found that VDD may be associated to longer ARI duration, but its effect on ARI incidence and its associated severity among healthy adults remains inconclusive. This review also highlighted the lack of a consistent regional and/or global definition for micronutrient sufficiency, and that future studies should explore and conclude the association between other micronutrient deficiency and ARIs in healthy adults before considering supplementation for ARI prevention and management.

## Introduction

Beneficial effects of micronutrients on the immune system have long been established and well documented in many studies [1–3]. Micronutrients work to maintain and strengthen one’s immune system through their action on epithelial barriers, cellular immunity and antibody production [1, 4]. However, approximately 2 billion people were estimated to suffer from micronutrient deficiency globally [4, 5]. Deficiencies commonly exist for zinc, iron, iodine and vitamins A, C and E, and are prevalent in pregnant women and children due to higher physiological demands [4–6]. Populations from developing countries and the aged populations are also prone to micronutrient deficiency due to undernutrition, or a lower dietary micronutrient density coupled with low use of dietary supplements [5, 7–9].

Micronutrients deficiencies are associated to varied adverse health outcomes, including a weakened immune system leading to higher risk for infections [1]. In the vulnerable population, the need of sufficient micronutrients for proper immune function is evident. A cross-sectional study in elderly Ecuadorian population showed that deficiency in folic acid, zinc, or vitamins C, D, B-6 or B-12 was associated to recent episodes of the common cold or pneumonia [10]. Lower zinc status was associated with increased susceptibility to infectious diseases such as malaria, diarrhea and acute lower respiratory infection in children and pregnant women [11]. Indeed, decreased acute respiratory infection (ARI) episodes and attenuation of its associated duration and severity have been observed when children were supplemented with zinc or vitamins E or A [4]. However, similar benefits on ARIs were absent when zinc or vitamins D or C supplements were given to a healthy population [2, 12] Nonetheless, a mild micronutrient deficiency can already cause negative effects on a normal adult’s immune system [13].

Despite the high economic burden for societies and impaired quality of life caused by ARIs, especially the common cold, in a healthy adult population [14, 15], no systematic review has ever assessed the effects of micronutrient deficiency on ARI, specifically the common cold in this population. Thus, this review aims to systematically search and summarise observations on the association between any single micronutrient deficiency and ARI incidence, duration and severity in healthy adults. We hypothesise that deficiency in any single micronutrient is likely to increase the risk for common ARI episodes and its associated duration and severity.

## Methods

### Study identification and selection

Four electronic databases (PubMed, Cochrane Library, Embase and Scopus) were searched in January 2019 to identify observational studies which measured micronutrient status in healthy adults infected with ARI. Specific search terms defined with the Population, Exposure, Outcome and Study Design (PEOS) criteria and strategies applied to each database are respectively provided in Additional file 1: Tables S1 and Additional file 1: Tables S2. To identify additional studies, reference lists of relevant reviews was searched and any year or language restrictions were not applied in the search strategy. However, only studies published in English were eventually selected as the reviewers were unable to accurately interpret the five potentially eligible non-English studies. This study was reported according to the Preferred Reporting Items for Systematic Reviews and Meta-Analyses group (PRISMA) [16].

Studies extracted from the database search were included when the following criteria were satisfied:

1. Types of studies: Observational studies which included a comparator group in the study design i.e. case-control, cross-sectional and cohort study designs, and were reported in English.
2. Type of participants: Healthy subjects without chronic conditions or comorbidities, or including some subjects with chronic conditions with the diseases statistically adjusted for in analyses i.e. a population with solely healthy or healthy and diseased subjects (mixed health status); a mean age of 18–65 years old. Subjects may be infected with a naturally-acquired ARI or community-acquired pneumonia (CAP).
3. Type of exposure: Deficiency in a single micronutrient of interest, including minerals (copper, iron, magnesium, selenium, zinc) and vitamins (Vitamins A, B, D, E and K), compared to a group without deficiency in the same micronutrient.
4. Types of outcome measures: Reported episodes, duration (in days) and/or severity scores of ARI (primary outcome) or CAP episodes (secondary outcome).

On hindsight, we also allowed the inclusion of studies which utilised a population with mixed health status, provided the outcomes reported adjusted for the included diseases. The micronutrients of interest in this review include minerals (copper, iron, magnesium, selenium, zinc) and vitamins (Vitamins A, B, D, E and K). This review defines an ARI episode as any upper respiratory tract infection, acute respiratory tract infection, influenza, influenza-like illnesses and common cold episodes, regardless whether the illness was clinically diagnosed, laboratory-confirmed or self-reported as defined by the study.

**Fig. 1:**
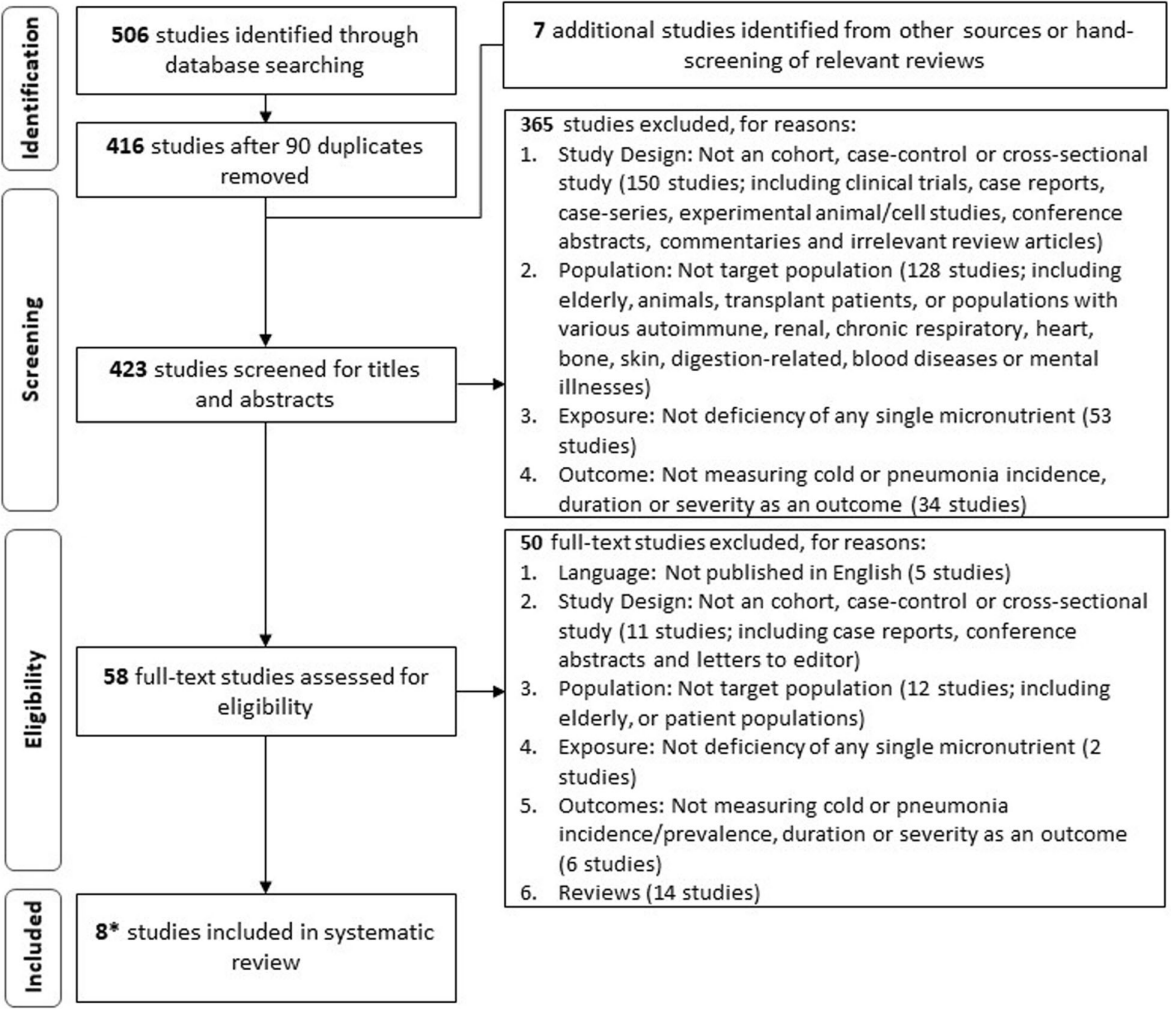
Flowchart of the process of study selection. *Only 4 studies clearly met the inclusion criteria [17–20]; the other 4 studies did not clearly meet the inclusion criteria due to missing information [21–24] but were judged to be relevant and highly likely to meet the inclusion criteria should missing information become available

The titles and abstracts of extracted studies were first screened for relevance; full texts of relevant studies were retrieved for further screening and validation according to the above criteria before final inclusion into the review. A PRISMA flow diagram and checklist of the study selection process are shown in Fig. 1 and Additional file 2, respectively.

### Data extraction

Data extracted from selected studies were summarised in Tables 1, 2 and 3. When clarification or more information were required, we also contacted corresponding authors of included studies. The following data were extracted from each study: authors, year of publication, study and population characteristics, the micronutrient measured in that study, cut-off concentration values for micronutrient categories (deficiency, inadequacy and sufficiency in nmol/L), and outcomes. In the case where concentrations of micronutrient categories were reported in ng/mL, they were converted to nmol/L using the conversion factor of 1 ng/mL = 2.5 nmol/L. We also assessed the study design of the included studies based on their design features as recommended by the Cochrane Handbook for Systematic Reviews and Meta-analyses, in addition to extracting their reported study design [25]. Outcome measures extracted for each micronutrient category, when available, include 1) the number of ARI episodes, 2) adjusted summary statistic for ARI incidence (relative risk (RR) or odds ratio (OR)), the median and interquartile ranges of 3) duration and 4) severity of common ARI episodes, and 5) any other key findings. The same outcome measures were also extracted for community-acquired pneumonia episodes.

**Table 1:** Characteristics of included studies

Author, Year [Ref.]	Reported Study Design	Country of study; follow-up duration	Population size and description (% Men); Mean Age (years);	Micronutrient in question	Cut-offs for different micronutrient categories, as defined by study (nmol/L)					Outcome reported
					Deficient	Inadequate	Sufficient	Optimal		
Met inclusion criteria clearly										
Laaksi, 2007 [19]	Prospective cohort	Finland; 6 months	756 Young Finnish military conscripts (100%); 18–29*	Vitamin D	<40	–	≥40	–	–	• Clinical RTI incidence • RTI duration
He, 2013 [17]	Prospective cohort	UK; 4 months	225 healthy endurance exercise athletes with ≥3 h of total moderate/high-intensity training per week (69.8%); 21	Vitamin D	12 to 30	30 to 50	50 to 120	≥120	–	• Self-reported upper RTI incidence • Self-reported upper RTI duration • Subjective symptom severity per episode
Jovanonich, 2014 [18]	Retrospective case-control	US; 3–15 months	132 patients hospitalised for pneumonia (cases) or other reasons (controls) (29%); 60	Vitamin D	< 37	37 to 50	51 to 75	> 75	–	• Laboratory-confirmed pneumonia incidence
Rafiq, 2018 [20]	Cross sectional	Netherlands; 1 month prior to interview	6138 men and women living in the greater area of Leiden with self-reported BMI ≥27 kg/m^2^ (43.9%); 55 (deficient), 55.9 (inadequate), 55.7 (sufficient)	Vitamin D	< 50	50 to 75	≥75	–	–	• Self-reported common cold incidence
Highly likely to meet inclusion criteria										
Sabetta, 2010 [24]	Prospective cohort	US; 4 months	198 healthy adults (42.9%); 20–88*	Vitamin D	< 95	–	≥95	–	–	• Clinical acute viral RTI incidence • Acute viral RTI duration
Berry, 2011 [21]	Cross sectional	UK; 3 weeks prior to interview	6789 non-pregnant free-living Caucasians born in England, Scotland and Wales during a week in March 1958 (birth cohort) (49.9%); 45	Vitamin D	< 25	25 to 49.9	50 to 74.9	75 to 99.9	≥100	• Self-reported influenza, pneumonia, bronchitis or severe cold incidence
Nanri, 2017 [23]	Nested Case-control	Japan; 6 months	532 employees from 4 companies in Kanto and Tokai regions (83.1%); 37.6	Vitamin D	< 50	50 to < 75	≥75	–	–	• Clinical influenza incidence
Lee, 2018 [22]	Retrospective cross sectional	US; 1 year post-vaccination	437 Young, healthy US military members vaccinated with 2009 monovalent influenza A (H1N1) vaccine (50.3%); Not reported by 90.6% of the population were 19–39 years old	Vitamin D	< 50	50 to 75	> 75	–	–	• Clinical influenza-like illness incidence

*RTI* Respiratory tract infections, *UK* United Kingdom, *US* United States of America, *age range

**Table 2:** Key findings on vitamin D deficiency on ARI incidence

Outcome	Author, Year	Population Health Status	Outcome ascertainment	Reported summary risk estimate: Odds Ratio (OR), Risk Ratio(RR) (95% Confidence Interval); % Population infected (infected/population size)				
				Deficient	Inadequate	Sufficient	Optimal	Per ↑ 10 nmol/L
Incidence of ARI/pneumonia	Laaksi, 2007 [19]	Healthy	Clinically diagnosed episodes of sinusitis, tonsillitis, otitis, bronchitis, pneumonia, pharyngitis, and laryngitis	1.03 (0.90, 1.18)^a,c^; 88.9% (24/27)	–	1.00 (reference); 86.1% (628/729)	–	–
	He, 2013 [17]	Healthy	Symptom diary; URTI was deemed present when (i) total symptom score was ≥15 on any two consecutive days and (ii) when a subject positively indicated suffering from a common cold on ≥3 days	NR; 66.7% (12/18)	NR; 39.7% (27/68)	NR; 43.8% (56/128)	NR; 27.3% (3/11)	–
	Jovanonich, 2014 [18]	Mixed^b^	Laboratory confirmed CAP with chest radiograph	2.57 (1.08, 6.08)^d^; NR	–	0.96 (0.35, 2.61)^d^; NR	1.03 (0.51, .09)^d^; NR	–
	Rafiq, 2018 [20]	Mixed^b^	Self-reported episodes of cold in the month prior to questionnaire	–	–	–	–	1.00 (0.96, 1.05)^e^; N/A
	Sabetta, 2010 [24]	Healthy	Self-reported, followed by clinical diagnosis of acute viral RTI, which may or may not be laboratory confirmed	1.00 (reference); 45.0% (81/180, 32 laboratory-confirmed cases)	–	–	0.52 (0.25, 0.84)^a^; 16.7% (3/18; 1 laboratory-confirmed case)	–
	Berry, 2011 [21]	Supposedly healthy	Self-reported episodes of respiratory infections (influenza, pneumonia, bronchitis, severe cold) in past 3 weeks prior to questionnaire	1.00 (reference)	0.94 (0.7, 1.27)^f^	0.74 (0.54, 1.02)^f^	75–99.9 nmol/L: 0.66 (0.46, 0.96)^f^; NR ≥100 nmol/L: 0.57 (0.34, 0.94)^e^; NR	0.93, (0.89, 0.97)^f^; N/A
	Nanri, 2017 [23]	Supposedly healthy	Self-reported to be clinically diagnosed with influenza, which may or may not be laboratory confirmed	1.00 (reference)^g^; 32.8% (59/180)	1.11 (0.74, 1.68)^g^; '28.6% (106/303)	0.77 (0.37,1.59)^g^; 28.6% (14/49)	–	–
	Lee, 2018 [22]	Healthy	Clinical diagnosis of ILI (retrospective retrieval from system)	1.43 (0.77, 2.67)^h^; NR	1.55 (0.84, 2.86)^h^; NR	1.00 (reference); NR	–	–

*RTI* Respiratory tract infections, *NR* Not Reported, *N/A* Not Applicable, *ILI* Influenza-like illness

^a^Relative risk reported, otherwise odds ratio was reported

^b^Mixed health status refers to populations that with healthy and diseased subjects with chronic diseases, but statistically adjusted for these diseases in analyses.

^c^Adjusted for smoking

^d^Adjusted for diabetes, renal disease and peripheral vascular disease; Odds are compared against the cut-offs for the following serum 25(OH)D levels in the respective categories, i.e. Deficient (<37 nmol/L vs ≥37 nmol/L), Sufficient (<50 nmol/L vs ≥50 nmol/L), optimal (<75 nmol/L vs ≥75 nmol/L)

^e^Adjusted for age, sex, ethnicity, number of packyears, self-reported obstructive pulmonary disease, use of pulmonary and anti-inflammatory medication, educational level, season, physical activity, BMI, total body fat and waist circumference

^f^Adjusted for gender, lifestyle factor, BMI and waist circumference

^g^Adjusted for influenza vaccination status, BMI, exercise and smoking statuses, living with schoolchildren, green tea intake and use of public bus or train for commuting

^h^Adjusted for age, gender, time from vaccination to serum collection

**Table 3:** Key findings on vitamin D deficiency on ARI duration and/or severity

Outcome	Author, Year	Population Health Status	Outcome ascertainment	Median duration/severity per episode (Interquartile range)			
				Deficient	Inadequate	Sufficient	Optimal
Duration of ARI/pneumonia episode	Laaksi, 2007 [19]	Healthy	Number of days absent from duty due to RTI	4 (2,6)	–	2 (0, 4)^a^	–
	He, 2013 [17]	Healthy	Total number of days with a symptom score of ≥5 in daily symptom diary	13 (10, 17)	8 (5, 14)^a^	8 (6, 9)^a^	5 (5,7)^a^
	Sabetta, 2010 [24]	Healthy	Symptom diary and follow-up by clinician every 1–3 days until asymptomatic	6 (2, 8)Percentage of days ill (days ill/days observed): 3.9% (777/19763)	–	6 (2, 27)Percentage of days ill (days ill/days observed): 0.80% (16/1994)	–
Severity of ARI/pneumonia episode	He, 2013 [17]	Healthy	Symptom diary; total symptom score for every subject each day was calculated as a sum of multiplied numbers of symptoms experienced by the numerical severity ratings (1 = mild, 2 = moderate, 3 = severe)	102 (67, 199)	62 (46, 74)^a^	47 (40, 69)^a^	43 (38, 52)^a^

^a^Significantly shorter than duration in deficient groups

### Quality assessment

Studies included in this review were assessed for their reporting quality and methodological quality using the methods described in Additional file 3. Reporting quality was assessed with the STrengthening the Reporting of OBservational studies in Epidemiology (STROBE) statement [26, 27]. The methodological quality was assessed with the National Lung, Heart, and Blood Institute (NLHBI) Quality Assessment Tool for Observational Cohort, Cross-sectional and Case-Control Studies [28]. The tool examines the risk of biases in the domains of selection, misclassification, detection, confounding, attrition and inappropriate sample sizes. The overall reporting and domain-specific methodological quality ratings given to each study were presented in Table 4, and a detailed breakdown of the assessment can be found in Additional file 3: Table S3.

**Table 4:** Reporting and methodological quality

Quality Assessed	Study Design	Cohort & Cross sectional Studies						Case-Control	
	Author, Year	Laaksi, 2007	He, 2013	Rafiq, 2018	Sabetta, 2010	Berry, 2011	Lee, 2018	Jovanovich, 2014	Nanri, 2017
	Section/ Domain								
Reporting Quality^a^	Title, Abstract and Introduction	High	Fair	High	Fair	High	High	High	High
	Methods	High	High	High	High	High	High	High	High
	Results	Low	High	Low	High	High	High	High	High
	Discussion	Fair	Low	High	High	High	High	High	High
	Other information	High	High	High	High	High	High	High	High
	Overall	Low	Fair	Fair	High	High	High	High	High
Methodology Quality^b^	Selection	High	High	High	High	High	High	High	High
	Misclassification	High	High	Low	High	High	Low	High	High
	Detection	Fair	Low	Low	High	Low	Low	High	High
	Confounding	Low	Low	High	Low	High	High	High	High
	Other (Inappropriate sample size, attrition)	Low	Low	High	Low	Low	Low	Low	Low

*Y* Yes, *N* No, *CT* Can’t tell, *N/A* Not Applicable, *P* Partially reported

^a^Assessed with the STROBE statement

^b^Assessed by the NHLBI Quality Assessment Tool for Observational Cohort, Cross-Sectional and Case-control studies; a high quality indicates a low risk of bias in the assessed domain, and vice versa

Study selection, data extraction and quality assessment were performed in duplicate by two independent reviewers. Differences in studies selected, data extracted or quality assessments between reviewers were discussed and resolved by consensus at the end of each procedure.

## Results

### Screening results and characteristics of included studies

Our literature searches identified 506 articles, of which 90 were duplicates. The articles covered various publication genres which were based on cells, animal and human populations. Of those based on human populations, most focused on elderly or patient populations with various diseases (Fig. 1). Exposures studied varied from the effects of a drug/treatment, to the presence of risk factors for a disease and the measurement of micronutrient levels in the study population. Micronutrients measured in the identified articles included copper, zinc, magnesium, manganese, rubidium, phosphorus, selenium, calcium and vitamins A, B, C, D. E and K. Following the screening process, 415 unique articles were excluded based on the inclusion criteria and eight articles were selected for final inclusion into our review. A flow chart of the screening process and the reasons for article exclusion are presented in Fig. 1. It was also noted that no articles were excluded as they assessed the effects of multi-micronutrients deficiency on our outcomes of interest. Of the eight articles included in this review, only four articles were clearly eligible for inclusion [17–20] while the other four articles did not clearly meet the inclusion criteria due to missing information [21–24]. Missing information from these studies include mean population age [22, 24] and an explicit indication of a healthy population used [21–23]. Nonetheless, these studies were deemed by the authors to be relevant and highly likely to meet the inclusion criteria should the missing information become available. Thus, these four studies were also included in the review.

The eight studies included in this review were mainly made up of Europeans and Americans (only one study with Japanese) and involved 15,207 adult participants (Table 1). Health status of included subjects varied, with 1616 confirmed healthy subjects from four studies [17, 19, 22, 24], 6270 subjects belonging to populations of mixed health status in two studies [18, 20] and 7321 subjects with supposedly healthy status due to limited descriptions in two other studies [21, 23]. The included studies reported a cohort [17, 19, 24], cross-sectional [20–22] or case-control [18, 23] design. Design features of all but one study matched their reported study design; the study had design features that suggested a retrospective cohort design although it reported a retrospective cross-sectional design [22]. Although we did not limit the micronutrients in the search and found a variety of micronutrients in the search results, the only micronutrient identified in the included studies was vitamin D in the form of serum 25-hydroxyvitamin D. The cut-off concentrations for the “deficiency”, insufficiency” and “sufficient” vitamin D groups ranged from 12 nmol/L to 95 nmol/L, 25 nmol/L to 75 nmol/L, and 40 nmol/L to 95 nmol/L respectively. Three studies were also observed to have an additional “Optimal” category, with a cut-off concentration between 75 nmol/L and 120 nmol/L [17, 18, 21], that was further stratified into subcategories at ≥100 nmol/L in 1 study [21]. Outcomes were clinically diagnosed and/or laboratory confirmed in five studies and self-reported in the remaining 3 studies.

### Outcomes

ARI incidence was reported in five studies [17, 19–21, 24], whilst one study reported on the incidence of CAP [18]. Odd ratios (ORs), relative risks (RRs) and the percentage of population infected with ARI reported for the various vitamin D categories suggested that ARI incidence was either not associated to, or associated with a slight increase in probability or risk of incidence when Vitamin D deficiency (VDD) was present (Table 2). Compared to the deficient group, reported ORs suggested that there was either no statistically significant difference in odds of ARI incidence (OR [95% CI]: 0.74 [0.54, 1.02] to 1.11 [0.74, 1.68]) or a 43% reduction (OR [95% CI]: 0.57 [0.34,0.94]) in the non-deficient groups [21, 23]. Nonetheless, a 48% lowered risk of ARI incidence was observed in the sufficient group (RR [95% CI]: 0.52 [0.25, 0.84], *p* < 0.001), compared to the deficient group [24]. However, when the sufficient group was the reference group, the relative risk reduction was no longer significant (RR [95% CI]: 1.03 [0.90, 1.18] to 1.55 [0.84, 2.86]) [19, 22]. During the follow-up period, a significantly bigger proportion of the deficiency group reported ARI episodes (28.3% [*p* = 0.015] to 39.4% [*p* = 0.039] higher) compared to the optimal group [17, 24]. However, there was no significant difference in the rate of ARI incidence between deficient and non-deficient groups (adjusted incidence rate ratio [95% CI]: 1.18 [0.988,1.40]) [19]. In addition, there was either a non-significant difference (OR [95% CI]: 1.00 [0.96, 1.05]) or a 7% reduction (OR [95% CI] 0.93 [0.89, 0.97], *p* = 0.001) on ARI incidence odds, with every 10 nmol/L increase in serum vitamin D levels [20, 21].

Across the non-deficient groups, i.e. those with insufficient, sufficient and optimal vitamin D status, conflicting associations between vitamin D status and ARI incidence were also observed. One study reported no difference in incidence between these groups (*p* = 0.062) [17], while another reported significantly different incidence (*p* < 0.001) and adjusted ORs (*p* = 0.015) across these groups [21].

In the study reporting on CAP incidence, increased likelihood for CAP incidence was associated with VDD, where a 157% increase in odds was observed in deficient group compared to the non-deficient groups (OR [95% CI]: 2.57 [1.08, 6.08]) [18].

Duration of the ARI episodes was reported in three studies, all of which consistently reported longer ARI durations or a higher percentage of total days ill in the deficient group compared to the non-deficient groups (median [IQR], total days ill (%): deficient group, 4 [2, 6] to 13 [10, 25], 3.9%; non-deficient group, 2 [0, 4] to 8 [5, 14], 0.80%) [17, 19, 24] (Table 3). The observations suggested that ARI duration was associated to vitamin D status, and that VDD could be associated with a longer ARI duration.

Severity of the ARI episode was only reported in one study [17]. The study reported significantly higher severity scores per ARI episode when in the deficient group compared to the non-deficient groups. The study also observed that the median severity score was significantly different across the deficient, insufficient, sufficient and optimal groups (*p* = 0.013), suggesting that vitamin D status is likely associated with ARI severity, but remained inconclusive.

### Reporting and methodological quality of included studies

Generally, most of the included studies had high overall reporting quality [18, 21–24]. The remaining two studies were judged with fair overall quality [17, 20] and only one study was judged with low overall quality [19] (Table 4). While the reporting quality of the title, abstract, introduction section were not included in the criteria for rating the overall reporting quality, they were generally well reported with a fair [17, 24] or high quality [18–23] (Additional file 3; Table S1). It was interesting to note that the included studies were generally well reported, though none explicitly stated adherence to the STROBE guidelines.

The eight studies were all of low risk of selection bias. All but two studies [20, 22] were at low risk of misclassification bias, although it was generally unclear across the cohort and the cross-sectional studies whether the follow-up timeframe was sufficient to assess the association between vitamin D deficiency (VDD) and the outcomes. Case-control [18, 23] and cross-sectional studies [20–22] respectively showed low and high risk of detection biases, and all adjusted for the key potential confounders identified statistically. In contrast, cohort studies included displayed varied risks of detection bias and a high risk of confounding bias [17, 19, 24].

## Discussion and conclusion

This review provides a complete overview of current literature investigating the association between deficiency in any single micronutrient and ARI, especially the common cold. The association assessment also included ARI-associated pneumonia incidence, duration and severity in a healthy population. To the best of our knowledge, this is the first review that attempts to systematically summarise the association between these variables in a healthy population. Nonetheless, the small number of studies included in this review and identification of vitamin D as the sole micronutrient studied for the outcomes of interest in our target population highlights the paucity of such studies in this population.

Although small in number, the included studies already showed mixed results on the association between VDD and ARI. The lack of association between these variables, to a slight increase in odds and risk of ARI or pneumonia with VDD persisted even when the findings were stratified according to health status of populations (confirmed healthy, mixed health status and supposedly healthy populations). This overlaps with observations in the vulnerable populations where VDD was generally associated with increased prevalence and/or incidence of respiratory diseases [29–31]. A meta-analysis revealed associations between low serum vitamin D levels and a higher risk of active tuberculosis in healthy adults (pooled effect size: 0.68, 95% C.I.: 0.43, 0.93) [32]. Another systematic review found high VDD prevalence amongst those suffering from chronic lung diseases such as asthma, cystic fibrosis and chronic obstructive pulmonary disease, and demonstrated the roles of VDD in the pathogenesis of these diseases [33]. While the causal relationship between VDD and respiratory disease incidence cannot be confirmed by randomised trials due to ethical reasons, studies have explored this plausible relationship between these variables through supplementation trials. The protective effect of vitamin D supplementation against respiratory infections have been consistently demonstrated in susceptible populations [2, 4, 7], hinting at the causality of VDD on ARI incidence. However, this effect is unclear in the generally healthy adult population [12, 34]. Nonetheless, a recent meta-analysis showed that vitamin D supplementation generally decreased the odds of an ARI episode in humans (OR [95% C.I.], 0.88 [0.81, 0.96]). This protective effect is even more pronounced when one has vitamin D levels below 25 nmol/L (OR [95% C.I.], 0.58 [0.40, 0.82]), but disappeared in all subgroups when the population was stratified by the presence of asthma or chronic obstructive pulmonary disease or age, except in the 1–16 years old age group (OR [95% C.I.], 0.60 [0.46, 0.77]) [34]. This observation supports the notion that a deficiency state may have more influence over ARI incidence (*p*-value for interaction _baseline 25-hydroxyvitamin D levels_ = 0.01) compared to an individual’s health status or age. The deficiency state may have a relatively larger impact than health or age on ARI incidence because vitamin D and its metabolites act as powerful immunoregulators, with most immune cells expressing vitamin D receptors. Thus, depleted serum vitamin D levels could have amplified effects on all downstream pathways triggered by vitamin D. This may expedite autoimmune development and compromise the immune system to a larger extent than sickness or aging [35]. Therefore, separating our population into the confirmed, mixed and supposedly healthy status still yielded mixed findings on the association between vitamin D status and ARI incidence as the health status has a relatively less pronounced effect on ARI incidence.

In this review, we developed an approach to summarise the sectional and overall reporting quality using the reporting qualities for the methods, results and discussion sections. The quality of the remaining sections assessed were omitted as they purely examine the reporting transparency and do not affect the risk of bias [36]. This is evident as the addition of those sections into the framework did not change the overall reporting quality assigned to all but one study (Additional file 3: Table S3) [19]. The absence of a valid approach to summarise the reporting qualities could be because the STROBE statement was intended to be a reporting guideline for observational studies. However, the use of the STROBE statement to assess reporting quality was not an inappropriate use of the guideline [36, 37]. The lack of a recommended quality assessment tool and approach to summarise the overall methodological quality of observational studies were also observed while performing this review. The Cochrane Handbook did recommend using the Newcastle-Ottawa Scale (NOS) to assess quality of non-randomised studies. However, we did not implement this tool as it was less comprehensive and rigorous than the NLHBI checklist used in this review. In addition, the NOS manual was not as clear and in-depth as that provided by NLHBI, resulting in difficulty in application of the tool. Nonetheless, the NLHBI checklist also lacked an approach to summarise the overall reporting quality. The current approach adopted in this review i.e. using the majority of rating in each domain (or the lower rating when there are 2 equal ratings for a domain) as the domain-specific quality, is conservative, simplistic and may overestimate the risk of bias in each domain. Nevertheless, it presents the risk of bias for each assessed domain in a transparent manner, and does not misuse the NLHBI checklist [38].

### Strengths, limitations and future work

A strength of this review was that it encompasses all relevant literature published since inception of the electronic databases searched, as there was no restriction on publication year in the search strategy. This ensured that this review presented a complete overview of all relevant research done in this area, in the healthy adult population. Another strength of this review was that it included a more complete quality assessment of the included studies. “Quality” in research refers to both methodological and reporting quality of a study [37]. However, only the former is generally assessed as part of the risk of biasness assessment for included studies in reviews. This could be because reporting quality assessment is not included as part of the recommended systematic reviews and meta-analysis protocol by the Cochrane Handbook [25]. Nonetheless, the reporting quality of a study can affect the ease of assessing methodological quality and hence, the confidence in the risk of bias generated for the study [37]. As all but one of the included studies [19] in this review had fair to high reporting quality, we can have increased confidence in the given overall methodological quality rating.

It is worth noting some limitations of this review. Firstly, a pooled risk estimate for the outcomes of interest cannot be generated, and hence cannot assess the certainty of evidence presented in this review. The inability to statistically analyse the evidence was due to missing data and the small number of studies reporting these outcomes. Furthermore, some of the studies were included although they did not clearly meet the inclusion criteria or had some diseased subjects. This further decreases credibility of the conclusion drawn although these studies were highly likely to meet the inclusion criteria, or the extracted study results were statistically adjusted for diseases if present. Secondly, 96.5% of the study populations were Caucasian, limiting the external validity of our findings as it may not represent the effects of VDD on a ARI incidence, duration and severity in a healthy Asian population. Due to a lack of studies investigating the effect of other micronutrients deficiency on the outcomes of interest, our findings are limited to only VDD. Furthermore, multi-micronutrient deficiencies were more commonly observed than single micronutrient deficiencies, further limiting the generalisabilty of our findings [5, 39]. Thirdly, publication bias may exist as we searched only within published literature and eventually only included those in English, although language was not restricted for in the search strategy. Moreover, although included studies generally adjusted for confounders, except for 3 studies that either partially explored [19] or did not explore confounding entirely [17, 24], residual confounding may still exist. Lastly, the observational nature of the studies limits our assessment to whether an association exists between VDD and the outcomes. Hence, we are unable to determine whether there is a direct or reverse causal relationship between these variables.

Nonetheless, this review highlighted the need to develop a consistent definition for “deficiency” and “sufficiency”, at least for each region to account for geographical, seasonal and ethnicity differences in populations from different regions. Varying cutoff concentrations for the vitamin D categories were observed in included studies, but US Institute of Medicine (IOM) and experts in the field suggested similar cutoff concentrations of 12 ng/mL and 12.5 ng/mL to define the “deficiency” group. This suggests a consistent increased risk for adverse health effects below these vitamin D levels [40, 41]. However, the same experts mentioned that the IOM cutoff for “sufficient” (≥20 ng/mL) was more applicable for populations most at risk for a true VDD, and overestimated the vitamin D levels required for normal cell and immune functions in healthy individuals (approximately 15 ng/mL) [41, 42]. Instead, unnecessary supplementation of healthy individuals with vitamin D levels between 12.5 to 20 ng/mL may result in toxicity. Toxicity occurs when vitamin D intake exceeds the tolerable upper intake level, resulting in vitamin D levels of about 50 ng/mL [40, 41]. In light of these considerations, the review authors propose cutoff concentrations of <12.5 ng/mL (31.25 nmol/L) for “deficient”, 12.5 to <50 ng/mL (31.25–125 nmol/L) for “sufficient” and > 50 ng/mL (>125 nmol/L) as “toxic” for healthy children and adults in the western regions. However, the proposed cutoff concentrations will not be applicable to the Asian region, and more VDD studies conducted in Asian populations would be required to establish those values. This review also highlighted the need for more studies on micronutrient deficiency on respiratory health, specifically ARIs, in a healthy adult population. This is required to substantiate and validate the need of supplementation, especially micronutrients shown to improve or maintain respiratory health but were excluded from this review as no studies investigated the effect of its deficiency on ARI incidence, duration or severity in a healthy adult population. More importantly, if there is a true association between micronutrient deficiency and ARI incidence, duration and/or severity, then micronutrient supplementation might be a viable and simple solution to improve overall well-being of healthy individuals whilst reducing public health burden.

In conclusion, the association between VDD and ARI incidence and severity remains inconclusive. However, VDD is likely to increase the duration of ARI especially common cold. More future studies are still required to strengthen these findings.

## Supplementary information

**Additional File 1. **Description of PICOS criteria for a systematic review and search strategy. **Table S1.** Description of PICOS criteria for a systematic review assessing the association of micronutrient deficiency with the incidence, duration and severity of common cold and pneumonia. **Table S2.** Search strategy for micronutrient deficiency of cold and/or pneumonia PubMED, Embase, Scopus and the Cochrane Library.

**Additional File 2.** PRISMA Checklist.

**Additional File 3. **Methods for methodological quality assessment & for reporting quality assessment. **Table S1.** Classification of risk of bias domains assessed with published questions in NLHBI quality assessment tool for observational cohort, cross-sectional and case-control studies. **Table S2.** Proposed framework for determining overall reporting quality based on title, abstract and introduction, methods, results and discussion sections. **Table S3.** Breakdown of methodological and reporting quality assessment ratings given for each included study.

## Data Availability

All data generated or analysed during this study are included in this published article and supplementary information files.

## References

[1] Maggini S, Wintergerst ES, Beveridge S, Hornig DH (2007). Selected vitamins and trace elements support immune function by strengthening epithelial barriers and cellular and humoral immune responses. Br J Nutr.

[2] Rondanelli M, Miccono A, Lamburghini S, Avanzato I, Riva A, Allegrini P, Faliva MA, Peroni G, Nichetti M, Perna S (2018). Self-care for common ARIs: the pivotal role of vitamin D, vitamin C, zinc, and Echinacea in three main immune interactive clusters (physical barriers, innate and adaptive immunity) involved during an episode of common ARIs—Practical advice on dosages and on the time to take these nutrients/botanicals in order to prevent or treat common ARIs. Evid-Based Complement Altern Med.

[3] Shenkin A (2006). Micronutrients in health and disease. Postgrad Med J.

[4] Katona P, Katona-Apte J (2008). The interaction between nutrition and infection. Clin Infect Dis.

[5] Bailey RL, West KP, Black RE (2015). The epidemiology of global micronutrient deficiencies. Ann Nutr Metab.

[6] Ritchie H, Roser M. Micronutrient deficiency. 2019. Retrieved from: https://ourworldindata.org/micronutrient-deficiency. Accessed 1 Sept 2019.

[7] Hwalla N, Al Dhaheri A, Radwan H, Alfawaz H, Fouda M, Al-Daghri N, Zaghloul S, Blumberg J (2017). The prevalence of micronutrient deficiencies and inadequacies in the Middle East and approaches to interventions. Nutrients.

[8] Beal T, Massiot E, Arsenault JE, Smith MR, Hijmans RJ (2017). Global trends in dietary micronutrient supplies and estimated prevalence of inadequate intakes. PLoS One.

[9] Conzade R, Koenig W, Heier M, Schneider A, Grill E, Peters A, Thorand B (2017). Prevalence and predictors of subclinical micronutrient deficiency in german older adults: results from the population-based KORA-age study. Nutrients.

[10] Hamer DH, Sempértegui F, Estrella B, Tucker KL, Rodríguez A, Egas J, Dallal GE, Selhub J, Griffiths JK, Meydani SN (2008). Micronutrient deficiencies are associated with impaired immune response and higher burden of respiratory infections in elderly Ecuadorians. J Nutr.

[11] Walker CF, Black RE (2004). Zinc and the risk for infectious disease. Annu Rev Nutr.

[12] Gysin DV, Dao D, Gysin CM, Lytvyn L, Loeb M (2016). Effect of vitamin D3 supplementation on respiratory tract infections in healthy individuals: a systematic review and meta-analysis of randomized controlled trials. PLoS One.

[13] PRASAD AS, RABBANI P, Abbasii A, BOWERSOX E, Fox MS (1978). Experimental zinc deficiency in humans. Ann Intern Med.

[14] Fendrick AM, Monto AS, Nightengale B, Sarnes M (2003). The economic burden of non–influenza-related viral respiratory tract infection in the United States. Arch Intern Med.

[15] Dicpinigaitis PV, Eccles R, Blaiss MS, Wingertzahn MA (2015). Impact of cough and common ARI on productivity, absenteeism, and daily life in the United States: ACHOO survey. Curr Med Res Opin.

[16] Moher D, Liberati A, Tetzlaff J, Altman DG (2009). Preferred reporting items for systematic reviews and meta-analyses: the PRISMA statement. Ann Intern Med.

[17] He CS, Handzlik M, Fraser WD, Muhamad A, Preston H, Richardson A, Gleeson M (2013). Influence of vitamin D status on respiratory infection incidence and immune function during 4 months of winter training in endurance sport athletes. Exerc Immunol Rev.

[18] Jovanovich AJ, Ginde AA, Holmen J, Jablonski K, Allyn RL, Kendrick J, Chonchol M (2014). Vitamin D level and risk of community-acquired pneumonia and sepsis. Nutrients.

[19] Laaksi I, Ruohola JP, Tuohimaa P, Auvinen A, Haataja R, Pihlajamaki H, Ylikomi T (2007). An association of serum vitamin D concentrations < 40 nmol/L with acute respiratory tract infection in young Finnish men. Am J Clin Nutr.

[20] Rafiq R, Thijs W, Prein R, de Jongh RT, Taube C, Hiemstra PS, de Mutsert R, den Heijer M (2018). Associations of serum 25 (OH) D concentrations with lung function, airway inflammation and common ARI in the general population. Nutrients.

[21] Berry DJ, Hesketh K, Power C, EJBJoN H (2011). Vitamin D status has a linear association with seasonal infections and lung function in British adults. Brit J Nutr.

[22] Lee RU, Won SH, Hansen C, Crum-Cianflone NF (2018). 25-hydroxyvitamin D, influenza vaccine response and healthcare encounters among a young adult population. PLoS One.

[23] Nanri A, Nakamoto K, Sakamoto N, Imai T, Akter S, Nonaka D, Mizoue T (2017). Association of serum 25-hydroxyvitamin D with influenza in case-control study nested in a cohort of Japanese employees. Clin Nutr.

[24] Sabetta JR, DePetrillo P, Cipriani RJ, Smardin J, Burns LA, Landry ML (2010). Serum 25-hydroxyvitamin d and the incidence of acute viral respiratory tract infections in healthy adults. PLoS One.

[25] HPage MJ, Higgins JPT, Sterne JAC. Chapter 13: Assessing risk of bias due to missing results in a synthesis. In: Higgins JPT, Thomas J, Chandler J, Cumpston M, Li T, Page MJ, Welch VA (editors). Cochrane Handbook for Systematic Reviews of Interventions version 6.0 (updated July 2019). Cochrane. 2019. Available from www.training.cochrane.org/handbook. Accessed 1 Sept 2019.

[26] Vandenbroucke JP, von Elm E, Altman DG, Gøtzsche PC, Mulrow CD, Pocock SJ, Poole C, Schlesselman JJ, Egger M, Initiative S (2014). Strengthening the reporting of observational studies in epidemiology (STROBE): explanation and elaboration. Int J Surg.

[27] Von Elm E, Altman DG, Egger M, Pocock SJ, Gøtzsche PC, Vandenbroucke JP (2007). The strengthening the reporting of observational studies in epidemiology (STROBE) statement: guidelines for reporting observational studies. Ann Intern Med.

[28] NHLBI Study Quality Assessment Tools [https://www.nhlbi.nih.gov/health-topics/study-quality-assessment-tools].

[29] Almario CV, Metz DC, Haynes K, Yang YX (2015). Risk of community-acquired pneumonia in patients with a diagnosis of pernicious anemia: a population-based retrospective cohort study. Eur J Gastroenterol Hepatol.

[30] Esposito S, Lelii M (2015). Vitamin D and respiratory tract infections in childhood. BMC Infect Dis.

[31] Hirani V (2013). Associations between vitamin D and self-reported respiratory disease in older people from a nationally representative population survey. J Am Geriatr Soc.

[32] Nnoaham KE, Clarke A (2008). Low serum vitamin D levels and tuberculosis: a systematic review and meta-analysis. Int J Epidemiol.

[33] Kokturk N, Baha A, Oh YM, Young Ju J, Jones PW (2018). Vitamin D deficiency: what does it mean for chronic obstructive pulmonary disease (COPD)? A compherensive review for pulmonologists. Clin Respir J.

[34] Martineau AR, Jolliffe DA, Hooper RL, Greenberg L, Aloia JF, Bergman P, Dubnov-Raz G, Esposito S, Ganmaa D, Ginde AA (2017). Vitamin D supplementation to prevent acute respiratory tract infections: systematic review and meta-analysis of individual participant data. Bmj.

[35] Hughes D, Norton R (2009). Vitamin D and respiratory health. Clin Exp Immunol.

[36] da Costa BR, Cevallos M, Altman DG, Rutjes AW, Egger M (2011). Uses and misuses of the STROBE statement: bibliographic study. BMJ Open.

[37] Harrison JK, Reid J, Quinn TJ, Shenkin SD (2016). Using quality assessment tools to critically appraise ageing research: a guide for clinicians. Age Ageing.

[38] Sanderson S, Tatt ID, Higgins J (2007). Tools for assessing quality and susceptibility to bias in observational studies in epidemiology: a systematic review and annotated bibliography. Int J Epidemiol.

[39] Abeywickrama H, Koyama Y, Uchiyama M, Shimizu U, Iwasa Y, Yamada E, Ohashi K, Mitobe Y (2018). Micronutrient status in Sri Lanka: a review. Nutrients.

[40] Vitamin D - Fact Sheet for Health Profesisonals [https://ods.od.nih.gov/factsheets/VitaminD-HealthProfessional/#en1].

[41] Manson J, Brannon PM, Rosen CJ, Taylor CL (2016). Vitamin D deficiency-is there really a pandemic?. N Engl J Med.

[42] Tello M. In: Tello M, editor. Vitamin D: What’s the “right level”. Harvard Health Blog; 2016. Available from https://www.health.harvard.edu/blog/vitamin-d-whats-right-level-2016121910893. Accessed 1 Sept 2019.

