# Supplementary material for: Association between micronutrient deficiency and acute respiratory infections in healthy adults: a systematic review of observational studies
Source: Nutr J. 2019 Nov 30;18:80. doi: 10.1186/s12937-019-0507-6 (PMC6885309; doi:10.1186/s12937-019-0507-6)
Supplement: Supplementary file 1 — Additional File 1. Description of PICOS criteria for a systematic review and search strategy. Table S1. Description of PICOS criteria for a systematic review assessing the association of micronutrient deficiency with the incidence, duration and severity of common cold and pneumonia. Table S2. Search strategy for micronutrient deficiency of cold and/or pneumonia PubMED, Embase, Scopus and the Cochrane Library. [file 12937_2019_507_MOESM1_ESM.docx]

**Additional File 1.**

**Table S1.** Description of PICOS criteria for a systematic review assessing the association of micronutrient deficiency with the incidence, duration and severity of common cold and pneumonia.

| **Variable** | **Description** |
| --- | --- |
| Population | Incidence: Healthy adults between 18 to 65 years old |
|  | Duration and severity: Healthy adults between 18 to 65 years old, infected with common cold or pneumonia |
| Exposure | Deficiency in any singular micronutrient.  Micronutrients of interest: minerals (copper, iron, magnesium, selenium, zinc) and vitamins (Vitamins A, B, D, E and K) |
| Comparator | Control groups without any micronutrient deficiency (for that single micronutrient of interest) |
| Outcome | Incidence, duration or severity of common cold (primary outcome) or community-acquired pneumonia (secondary outcome) |
| Study Design | Case-control, cross-sectional and cohort studies |
| Research Question | Is deficiency in any micronutrient associated with (i) common cold incidence/ prevalence, or (ii) when infected, cold duration or severity among healthy individuals aged between 18 and 65 years old? |

^1^ PICOS, Population, Intervention, Comparator, Outcome, Study design.

**Table S2**. Search strategy for micronutrient deficiency of cold and/or pneumonia PubMED, Embase, Scopus and the Cochrane Library.

| **Database** | **Search terms** | **PICOS criteria** | **Results yielded** |
| --- | --- | --- | --- |
| PubMED | (adult NOT (child* OR Infan*) | Population |  |
|  | "Magnesium deficiency"[MeSH] OR "Avitaminosis"[MeSH])) OR (((deficiency[subheading] OR deficien*[Title/Abstract])) AND (((((Micronutrients[MeSH] OR "micronutrient*"[Title/Abstract])) OR (Minerals[MeSH] OR mineral*[Title/Abstract])) OR ("Zinc Compounds"[Mesh] OR "Zinc"[Title/Abstract] OR "Selenium"[Title/Abstract] OR "Selenite"[Title/Abstract] OR "Selenate"[Title/Abstract] OR "Selenocysteine"[Title/Abstract] OR "Selenomethionine"[Title/Abstract] OR "selenium yeast" OR "selenium enriched yeast" OR "magnesium"[Title/Abstract] OR "Copper"[Title/Abstract] OR "cupric"[Title/Abstract] OR "iron"[Title/Abstract])) OR ("Vitamin *"[Title/Abstract] OR "ergocalciferol"[Title/Abstract] OR "cholecalciferol"[Title/Abstract] OR "dihydrotachysterol"[Title/Abstract]) OR "beta carotene"[Title/Abstract] OR "tocopherol"[Title/Abstract] OR "tocotrienol"[Title/Abstract] OR "Thiamine"[Title/Abstract] OR "Riboflavin"[Title/Abstract] OR "Niacinamide"[Title/Abstract] OR "Pantothenic acid"[Title/Abstract] OR "Biotin"[Title/Abstract] OR "Folic acid"[Title/Abstract] OR "ascorbic acid"[MeSH] OR "ascorb*"[Title/Abstract] OR "cevitamic acid"[Title/Abstract])) | Exposure |  |
|  | (("Pneumonia"[Mesh] AND "Community-Acquired Infections"[Mesh]) OR "Respiratory Tract Infections"[Mesh] OR "Common cold"[Mesh] OR "Community-acquired Pneumonia" OR "respiratory tract infections" OR "Respiratory infections" OR "common cold" OR "human influenza"[MeSH] OR influenza? OR flu) NOT tuberculosis) | Outcome |  |
|  | - | Study Design | 82 |
|  | PEOS | | 194 |
| Embase | 'adult'/exp OR 'adult' OR 'adults' OR 'grown-ups' OR 'grownup' OR 'grownups') NOT ('child'/exp OR 'child' OR 'children' | Population | 0 |
|  | 'iron deficiency'/exp OR 'deficiency, iron' OR 'fe deficiency' OR 'iron deficiency' OR 'sideropenia' OR 'magnesium deficiency'/exp OR 'deficiency, magnesium' OR 'magnesium deficiency' OR 'mg deficiency' OR 'mineral deficiency'/exp OR 'deficiency, mineral' OR 'mineral deficiency' OR 'selenium deficiency'/exp OR 'deficiency, selenium' OR 'selenium deficiency' OR 'zinc deficiency'/exp OR 'zinc deficiency' OR 'zn deficiency' OR 'vitamin deficiency'/exp OR 'avitaminosis' OR 'avitaminosis b' OR 'choline deficiency' OR 'corrinoid deficiency' OR 'deficiency, vitamin' OR 'hypovitaminosis' OR 'multiavitaminosis b' OR 'vitamin b complex deficiency' OR 'vitamin b deficiency' OR 'vitamin deficiency' OR 'copper deficiency'/exp OR 'copper deficiency' OR 'cu deficiency' OR 'deficiency, copper') NOT ('pellagra'/exp OR 'italian leprosy' OR 'lombardy leprosy' OR 'maidism' OR 'pellagra' OR 'pellagra dermatitis' OR 'pellagrous skin' OR 'proton pump inhibitor'/exp OR 'gastric proton pump inhibitor' OR 'hydrogen potassium adenosine triphosphatase inhibitor' OR 'hydrogen potassium atpase inhibitor' OR 'proton pump inhibitor' OR 'proton pump inhibitors' | Intervention |  |
|  | ('common cold'/exp OR 'cold, common' OR 'common cold' OR 'common colds' OR 'coryza' OR 'natural cold' OR 'natural colds' OR 'community acquired pneumonia'/exp OR 'community acquired pneumonia' OR 'viral respiratory tract infection'/exp OR 'respiratory tract infection, viral' OR 'respiratory tract viral infection' OR 'respiratory tract virus infection' OR 'respiratory viral infection' OR 'viral respiratory disease' OR 'viral respiratory tract infection' OR 'virus respiratory tract infection' OR 'influenza'/exp OR 'flu' OR 'flue' OR 'influenza' OR 'influenza infection' OR 'influenza syndrome' OR 'influenza, human | Outcome |  |
|  | - | Study Design |  |
|  | PEOS | | 152 |
| Scopus | TITLE-ABS-KEY ( adult )  AND NOT  TITLE-ABS-KEY ( child  OR  infan* OR pregnan*) | Population |  |
|  | TITLE-ABS-KEY ( deficien* ) )  W/2  ( TITLE-ABS-KEY ( micronutrient )  OR  TITLE-ABS-KEY ( mineral )  OR  TITLE-ABS-KEY ( "vitamin *" )  OR  TITLE-ABS-KEY ( ascorb* )  OR  TITLE-ABS-KEY ( "cevitamic acid" )  OR  TITLE-ABS-KEY ( ergocalciferol )  OR  TITLE-ABS-KEY ( cholecalciferol )  OR  TITLE-ABS-KEY ( dihydrotachysterol )  OR  TITLE-ABS-KEY ( "beta carotene" )  OR  TITLE-ABS-KEY ( tocopherol )  OR  TITLE-ABS-KEY ( tocotrienol )  OR  TITLE-ABS-KEY ( thiamine )  OR  TITLE-ABS-KEY ( riboflavin )  OR  TITLE-ABS-KEY ( niacinamide )  OR  TITLE-ABS-KEY ( "Pantothenic acid" )  OR  TITLE-ABS-KEY ( biotin )  OR  TITLE-ABS-KEY ( "folic acid" )  OR  TITLE-ABS-KEY ( zinc )  OR  TITLE-ABS-KEY ( selen* )  OR  TITLE-ABS-KEY ( magnesium )  OR  TITLE-ABS-KEY ( copper )  OR  TITLE-ABS-KEY ( iron ) | Intervention |  |
|  | TITLE-ABS-KEY ( ( community  W/2  acquired )  W/2  pneumonia )  OR  TITLE-ABS-KEY ( respiratory  W/2  infection )  OR  TITLE-ABS-KEY ( "common cold" ) ) AND NOT  ((TITLE-ABS-KEY ( trial ) OR TITLE-ABS-KEY ( tuberculosis )) | Outcome, Study Design |  |
|  | PEOS | | 160 |

| **Database** | **Search ID** | **Search terms** | **PICOS criteria** | **Results yielded** |
| --- | --- | --- | --- | --- |
| Cochrane Library | #1 | MeSH descriptor: [Adult] explode all trees | Population |  |
|  | #2 | MeSH descriptor: [Young Adult] explode all trees |  |  |
|  | #3 | MeSH descriptor: [Middle Aged] explode all trees |  |  |
|  | #4 | MeSH descriptor: [Child] explode all trees |  |  |
|  | #5 | MeSH descriptor: [Aged] explode all trees |  |  |
|  | #6 | (#1 and #2 and #3) not (#4 or #5) |  |  |
|  | #7 | MeSH descriptor: [Pneumonia] explode all trees | Outcome |  |
|  | #8 | MeSH descriptor: [Community-Acquired Infections] explode all trees |  |  |
|  | #9 | #7 and #8 |  |  |
|  | #10 | MeSH descriptor: [Common Cold] explode all trees |  |  |
|  | #11 | MeSH descriptor: [Influenza, Human] explode all trees |  |  |
|  | #12 | MeSH descriptor: [Respiratory Tract Infections] explode all trees |  |  |
|  | #13 | (common cold):ti,ab,kw or (community-acquired pneumonia):ti,ab,kw or (influenza*):ti,ab,kw or (flu):ti,ab,kw or (respiratory tract infection?):ti,ab,kw or (acute respiratory infection?):ti,ab,kw |  |  |
|  | #14 | #9 or #10 or #11 or #12 or #13 |  |  |
|  | #15 | (deficien*):ti,ab,kw | Intervention |  |
|  | #16 | MeSH descriptor: [Micronutrients] explode all trees |  |  |
|  | #17 | (Vitamin):ti,ab,kw OR (mineral*):ti,ab,kw OR (zinc):ti,ab,kw OR (selen*):ti,ab,kw OR (magnesium):ti,ab,kw OR (copper):ti,ab,kw OR (cupr*):ti,ab,kw OR (iron):ti,ab,kw OR (ferric):ti,ab,kw OR (ferrous):ti,ab,kw OR (ferrochel):ti,ab,kw |  |  |
|  | #18 | #15 and (#16 or #17) |  |  |
|  | #19 | MeSH descriptor: [Avitaminosis] explode all trees |  |  |
|  | #20 | MeSH descriptor: [Magnesium Deficiency] explode all trees |  |  |
|  | #21 | #18 or #19 or #20 |  |  |
|  | #22 | (supplement*):ti,ab,kw |  |  |
|  | #23 | #21 not #22 |  |  |
|  | #24 | #6 and #13 and #23 | PEOS | 0 |
