# Supplementary material for: Association between micronutrient deficiency and acute respiratory infections in healthy adults: a systematic review of observational studies
Source: Nutr J. 2019 Nov 30;18:80. doi: 10.1186/s12937-019-0507-6 (PMC6885309; doi:10.1186/s12937-019-0507-6)
Supplement: Supplementary file 2 — Additional File 2. PRISMA Checklist. [file 12937_2019_507_MOESM2_ESM.docx]

Additional File 3

*Methods for methodological quality assessment*

The methodological quality was assessed with the National Lung, Heart, and Blood Institute (NLHBI) Quality Assessment Tool for Observational Cohort, Cross-sectional and Case-Control Studies [1]. The tool was used to appraise the risk of bias in the domains of selection, misclassification, detection, confounding, attrition and inappropriate sample sizes for each study. Each question was answered using the guideline mentioned in the website, which did not mention an approach to summarise the methodological qualities of studies. Thus, the review authors grouped questions into the identified bias domains (Table B1, colour coded in Table B2) and assigned the domain-specific quality using the majority of the responses to questions in that domain. A majority of “yes”, “can’t tell” and “no” responses for questions in the domain corresponded to high, fair and low quality for the domain. In the case of a split response i.e. equal number of differing responses in the domain, the lower quality is assigned as the domain-specific quality for a conservative judgement.

The review authors did attempt to generate an overall methodological quality for each study by assigning the lowest quality given across all assessed domains as the overall methodological quality. This proposed approach assumed that all domains contributed equally to the overall quality, but also resulted in the unequal weighting of questions in the tool. This is especially so for domains with only one question assessing the risk of bias in that domain e.g. other bias and confounding domains. This approach may then be flawed as the overall methodological quality could potentially be dependent on the response to a single question. Thus, the proposed approach to summarise the overall methodological quality using the lowest domain-specific quality may not be robust as it could not convey the methodological quality of a study concisely but transparently. Therefore, the review authors did not generate an overall methodological quality for each study, but only reported the domain-specific qualities for each study to give a more accurate reflection of the methodological quality, and hence the risk of biases for each study (Table B3).

**Table S1**. Classification of risk of bias domains assessed with published questions in NLHBI quality assessment tool for observational cohort, cross-sectional and case-control studies.

| **Domain of Bias Assessed** | **Cohort & Cross sectional Studies** | **Case-control studies** |
| --- | --- | --- |
| Selection | Questions 2 - 4 | Questions 2, 4 - 8 |
| Misclassification | Questions 6 - 10 | Questions 9 - 10 |
| Detection | Questions 11, 12 | Question 11 |
| Confounding | Question 14 | Question 12 |
| Other (Inappropriate sample size, attrition) | Questions 5, 13 | Question 3 |

.

*Methods for reporting quality assessment*

Reporting quality was assessed with the STrengthening the Reporting of OBservational studies in Epidemiology (STROBE) statement [2, 3]. The statement was utilised as a checklist with “yes”, “no” and “partially” given as responses when questions were applicable to the given study design. “Yes” was given when the element raised by the question was reported in the study, “no” when the element is absent from the study and “partially” when the element was only partially reported in the study. The review authors then summarised the quality for each section using the majority of responses for that given section. A majority of “yes”, “partially” and “no” responses for questions in the section corresponded to high, fair and low quality for that given section. In the case of a split response i.e. equal number of differing responses in the section, the lower quality is assigned as the quality of the section for a conservative judgement. The review authors then used the quality of the methods, results and discussion sections to generate an overall reporting quality for the study, as these sections were deemed as the core sections in a publication. The title, abstract and introduction sections were not included in the framework to determine overall reporting quality as they do not significantly affect the overall reporting qualities of studies included, even when taken into consideration as shown in the results (Table B3). The proposed frameworks for assigning overall reporting quality based on the quality of the methods, results and discussion section of each study is shown in Table B2 (A), and that including the title, abstract and introduction sections is shown in Table B2(B).

**Table S2**. Proposed framework for determining overall reporting quality based on title, abstract and introduction, methods, results and discussion sections

| **Overall quality** | **Quality of section** | |
| --- | --- | --- |
| Sections considered in framework | Methods, Results and Discussion (A) | Title and abstract, Introduction, Methods, Results and Discussion (B) |
| High | All M, R, D = high | All I, M, R, D = high |
|  | 2 of M, R, D = high  1 of M, R, D = fair | 3 of I, M, R, D = high  1 of I, M, R, D = fair |
|  |  | 2 of I, M, R, D = high  2 of I, M, R, D = fair |
| Fair | All M, R, D = fair | All I, M, R, D = fair |
|  | 2 of M, R, D = high  1 of M, R, D = low | 3 of I, M, R, D = high  1 of I, M, R, D = low |
|  |  | 1 of I, M, R, D = high  3 of I, M, R, D = fair |
|  | 1 of M, R, D = high  2 of M, R, D = fair | 2 of I, M, R, D = high  2 of I, M, R, D = low |
|  |  | 2 of I, M, R, D = high  1 of I, M, R, D = fair  1 of I, M, R, D = low |
| Low | All M, R, D = low | 3 of I, M, R, D = fair  1 of I, M, R, D = low |
|  | 1 of M, R, D = high 1 of M, R, D = fair 1 of M, R, D = low | 1 of I, M, R, D = high  3 of I, M, R, D = low |
|  |  | 1 of I, M, R, D = high 2 of I, M, R, D = fair 1 of I, M, R, D = low |
|  | 1 of M, R, D = high 2 of M, R, D = low | 1 of I, M, R, D = high 1 of I, M, R, D = fair  2 of M, R, D = low |
|  | 2 of M, R, D = fair 1 of M, R, D = low | ≤2 of I, M, R, D = fair ≥2 of I, M, R, D = low |

I, Title, Abstract and Introduction; M, Methods; R, Results; D, Discussion

**Table S3.** Breakdown of methodological and reporting quality assessment ratings given for each included study.

|  | Study Design | Cohort & Cross sectional Studies | | | | | | Case-Control | |
| --- | --- | --- | --- | --- | --- | --- | --- | --- | --- |
| Methodology Quality^1^ | Author,  Year  Question | Laaksi, 2007 | He, 2013 | Rafiq, 2018 | Sabetta, 2010 | Berry, 2011 | Lee, 2018 | Jovanovich, 2014 | Nanri, 2017 |
|  | 1 | Y | Y | Y | Y | Y | Y | Y | Y |
|  | 2 | Y | Y | Y | Y | Y | Y | Y | Y |
|  | 3 | CT | CT | CT | CT | Y | CT | N | N |
|  | 4 | Y | Y | Y | Y | Y | Y | Y | Y |
|  | 5 | N | N | Y | Y | N | N | Y | Y |
|  | 6 | Y | Y | N | Y | N | N | Y | Y |
|  | 7 | CT | CT | CT | CT | Y | CT | Y | Y |
|  | 8 | Y | Y | Y | Y | Y | Y | N | Y |
|  | 9 | Y | Y | N | Y | Y | Y | Y | Y |
|  | 10 | N | Y | N | Y | N | N | Y | Y |
|  | 11 | Y | Y | N | Y | N | Y | Y | Y |
|  | 12 | CT | N | Y | Y | CT | N | Y | Y |
|  | 13 | Y | Y | N/A | N | N/A | N/A |  |  |
|  | 14 | N | N | Y | N | Y | Y |  |  |
|  | Selection | High | High | High | High | High | High | High | High |
|  | Misclassification | High | High | Low | High | High | Low | High | High |
|  | Detection | Fair | Low | Low | High | Low | Low | High | High |
|  | Confounding | Low | Low | High | Low | High | High | High | High |
|  | Other (Inappropriate sample size, attrition) | Low | Low | High | Low | Low | Low | Low | Low |
|  | Overall | Low | Low | Low | Low | Low | Low | Low | Low |
| Reporting Quality^2^ | 1a | N | Y | Y | Y | Y | P | N | Y |
|  | 1b | Y | P | Y | P | Y | Y | Y | Y |
|  | 2 | P | P | Y | Y | Y | Y | Y | Y |
|  | 3 | Y | P | Y | P | Y | Y | Y | Y |
|  | Reporting quality for Title, Abstract and Introduction | High | Fair | High | Fair | High | High | High | High |
|  | 4 | Y | Y | P | Y | Y | P | P | Y |
|  | 5 | P | Y | Y | P | Y | Y | Y | Y |
|  | 6a | P | Y | Y | P | Y | Y | Y | Y |
|  | 6b | Y | N/A | N/A | N/A | N/A | N/A | Y | Y |
|  | 7 | Y | P | Y | Y | Y | Y | Y | Y |
|  | 8 | Y | Y | Y | Y | Y | Y | Y | Y |
|  | 9 | N | N | Y | Y | N | Y | N | Y |
|  | 10 | N | Y | Y | Y | Y | N | Y | Y |
|  | 11 | P | P | Y | Y | Y | P | Y | Y |
|  | 12a | Y | Y | Y | Y | Y | Y | Y | Y |
|  | 12b | N | N | Y | Y | Y | N | N | P |
|  | 12c | Y | Y | Y | N | Y | Y | Y | Y |
|  | 12d | Y | Y | Y | N | N | N | Y | Y |
|  | 12e | N | N | Y | N | N | N | N | Y |
|  | Reporting quality for Methods | High | High | High | High | High | High | High | High |
|  | 13a | P | P | P | P | Y | P | Y | Y |
|  | 13b | N | Y | N | N | N | Y | Y | Y |
|  | 13c | N | N | N | N | N | N | N | Y |
|  | 14a | Y | Y | Y | Y | Y | Y | Y | Y |
|  | 14b | N | N | N | Y | P | Y | Y | P |
|  | 14c | N | N | N/A | N | N/A | N/A | N/A | N/A |
|  | 15 | P | Y | Y | Y | Y | Y | Y | Y |
|  | 16a | Y | N | P | Y | Y | Y | Y | Y |
|  | 16b | Y | Y | Y | Y | Y | Y | Y | Y |
|  | 16c | N | Y | N | P | Y | N | Y | N |
|  | 17 | N | Y | Y | Y | Y | N | N | Y |
|  | Reporting quality for Results | Low | High | Low | High | High | High | High | High |
|  | 18 | P | Y | Y | Y | Y | Y | Y | Y |
|  | 19 | P | N | P | Y | P | P | P | P |
|  | 20 | P | Y | Y | Y | Y | Y | Y | Y |
|  | 21 | N | N | N | Y | Y | Y | N | Y |
|  | Reporting quality for Discussion | Fair | Low | High | High | High | High | High | High |
|  | 22 | Y | Y | Y | Y | Y | Y | Y | Y |
|  | Reporting quality for Other information | High | High | High | High | High | High | High | High |
|  | Overall reporting quality (Using qualities for methods, results & discussion) | Low | Fair | Fair | High | High | High | High | High |
|  | Overall reporting quality (Using qualities for title, abstract & introduction, methods, results & discussion) | Fair | Fair | Fair | High | High | High | High | High |

1. **NHLBI Study Quality Assessment Tools** [<https://www.nhlbi.nih.gov/health-topics/study-quality-assessment-tools>]

2. Vandenbroucke JP, von Elm E, Altman DG, Gøtzsche PC, Mulrow CD, Pocock SJ, Poole C, Schlesselman JJ, Egger M, Initiative S: **Strengthening the Reporting of Observational Studies in Epidemiology (STROBE): explanation and elaboration.** *International journal of surgery* 2014, **12:**1500-1524.

3. Von Elm E, Altman DG, Egger M, Pocock SJ, Gøtzsche PC, Vandenbroucke JP: **The Strengthening the Reporting of Observational Studies in Epidemiology (STROBE) statement: guidelines for reporting observational studies.** *Annals of internal medicine* 2007, **147:**573-577.
